# Computational identification of *Vernonia cinerea*-derived phytochemicals as potential inhibitors of nonstructural protein 1 (NSP1) in dengue virus serotype-2

**DOI:** 10.3389/fphar.2024.1465827

**Published:** 2024-10-15

**Authors:** Md. Shohel Hossain, Soharth Hasnat, Shilpy Akter, Maria Mulla Mim, Anika Tahcin, Majedul Hoque, Durjoy Sutradhar, Mst. Alifa Akter Keya, Namin Rouf Sium, Sophia Hossain, Runa Masuma, Sakhawat Hossen Rakib, Md. Aminul Islam, Tofazzal Islam, Prosun Bhattacharya, M. Nazmul Hoque

**Affiliations:** ^1^ Department of Pharmacy, Jahangirnagar University, Dhaka, Bangladesh; ^2^ Molecular Biology and Bioinformatics Laboratory, Department of Gynecology, Obstetrics and Reproductive Health, Bangabandhu Sheikh Mujibur Rahman Agricultural University, Gazipur, Bangladesh; ^3^ Institute of Biotechnology and Genetic Engineering, Bangabandhu Sheikh Mujibur Rahman Agricultural University, Gazipur, Bangladesh; ^4^ Department of Pharmacy, Comilla University, Comilla, Bangladesh; ^5^ Department of Biochemistry and Molecular Biology, Noakhali Science and Technology University, Noakhali, Bangladesh; ^6^ Department of Pharmacy, ASA University Bangladesh, Dhaka, Bangladesh; ^7^ Electrical and Electronic Engineering, University of Asia Pacific, Dhaka, Bangladesh; ^8^ Advanced Molecular Lab, Department of Microbiology, President Abdul Hamid Medical College, Karimganj, Bangladesh; ^9^ COVID-19 Research, Department of Sustainable Development, Environmental Science and Engineering, KTH Royal Institute, Stockholm, Sweden

**Keywords:** DENV-2, NSP1, *V. cinerea*, molecular screening, molecular dynamics simulation

## Abstract

**Background:**

Dengue virus (DENV) infection, spread by *Aedes aegypti* mosquitoes, is a significant public health concern in tropical and subtropical regions. Among the four distinct serotypes of DENV (DENV-1 to DENV-4), DENV-2 is associated with the highest number of fatalities worldwide. However, there is no specific treatment available for dengue patients caused by DENV-2.

**Objective:**

This study aimed to identify inhibitory phytocompounds *in silico* in *Vernonia cinerea* (*V. cinerea*), a widely used traditional medicinal plant, for treating DENV-2 associated illnesses.

**Methods:**

The chemical structures of 17 compounds from *V. cinerea* were sourced from the Indian Medicinal Plants, Phytochemistry, and Therapeutics (IMPPAT) database. These compounds underwent geometry optimization, were screened against nonstructural protein 1 (NSP1) of DENV-2, and further validated through molecular dynamics simulations (MDS). Baicalein, an established drug against DENV-2, was used for validation in molecular screening, MDS, and MM-GBSA analyses.

**Results:**

Among these compounds, Beta-amyrin, Beta-amyrin acetate, Chrysoeriol, Isoorientin, and Luteolin showed promising potential as inhibitors of the NSP1 of DENV-2, supported by the results of thermodynamic properties, molecular orbitals, electrostatic potentials, spectral data and molecular screening. Besides, these compounds adhered to the Lipinski’s “rule of 5”, showing no hepatotoxicity/cytotoxicity, with mixed mutagenicity, immunotoxicity, and carcinogenicity. Furthermore, final validation through MDS confirmed their potential, demonstrating stable tendencies with significant inhibitory activities against NSP1 of DENV-2 over the control drug Baicalein. Among the screened compounds, Chrysoeriol emerged as the most promising inhibitor of NSP1 of DENV-2, followed by Luteolin and Isoorientin.

**Conclusion:**

Taken together, our results suggest that Chrysoeriol is the best inhibitor of NSP1 of DENV-2, which could be evaluated as a therapeutic agent or a lead compound to treat and manage DENV-2 infections.

## 1 Introduction

The dengue virus (DENV) belongs to the genus *Orthoflavivirus* within the family *Flaviviridae* (Postler et al., 2023). This genus also includes other viruses transmitted by mosquitoes and ticks that cause human diseases (Guzman et al., 2010; Guzman and Vazquez, 2010). The DENV crisis is significantly impacting global healthcare, with recent reports indicating over 100 million illnesses and 25,000 deaths attributed to dengue each year (Schaefer et al., 2020; Mohapatra et al., 2022). DENV is primarily transmitted to humans through the bites of infected female mosquitoes, mainly of the species *Aedes aegypti* (*A. aegypti*) and, to a lesser extent, *A. albopictus* (Ferreira-de-Lima and Lima-Camara, 2018). The DENV was first isolated in 1943, with DENV-1 being the earliest identified serotype (Normile, 2013). Dengue infections are caused by four closely related virus: DENV-1, DENV-2, DENV-3, and DENV-4, which share about 65% of their genomes (Normile, 2013). Despite this genetic similarity, there is still considerable genetic variation within each serotype (Normile, 2013; Khetarpal and Khanna, 2016). Among the serotypes, DENV-2 played a significant role in dengue-related fatalities, characterized by its distinct antigenic properties yet sharing similarities with other types (Vaughn et al., 2000). The genetic composition of DENV-2 is composed of an 11-kilobase RNA molecule (Murugesan and Manoharan, 2020). In addition to the open-reading frame (ORF), the viral RNA is surrounded by 5′- and 3′-untranslated regions (UTRs) that are crucial to translation, packaging, and replication (Wei et al., 2009). The genomic constituent of DENV-2 encodes around seven nonstructural proteins (NSP1 to NSP5) and three structural proteins such as envelope (E), membrane (M), and capsid (C) protein (Shrivastava et al., 2020). These components constitute the outer shell of the DENV-2, crucial for interacting with host cells and evading immune responses (da Fonseca et al., 2017; Verhaegen and Vermeire, 2024). Each of the seven NSPs of the DENV has a unique function. NSP1 is involved in viral replication and immune evasion, while NSP2A plays a role in viral assembly and RNA replication (Dey et al., 2021). NSP2B acts as a cofactor for NSP3 (Dey et al., 2021), which is essential for protease activity and crucial for viral replication. NSP4A facilitates host cell membrane rearrangement and viral replication, while NSP4B contributes to the formation of the viral replication complex and modulates host immune responses (Cortese et al., 2021). NSP5, the largest nonstructural protein, functions as an RNA-dependent RNA polymerase and methyltransferase, vital for viral RNA synthesis and capping (Li and Kang, 2022; Verhaegen and Vermeire, 2024). Given the virulence and biological significance of the seven NSPs of DENV-2, NSP1 stands out as a promising therapeutic target and vaccine candidate (Chen et al., 2018). Moreover, the experimental structure of NSP1 has been determined, highlighting its critical role in DENV-2 (Akey et al., 2014).

Despite the severe consequences and high mortality rate of DENV-2 infections, specific treatment options beyond symptomatic relief measures remain lacking. This challenge stems from various factors, including the rapid replication of the DENV-2, frequent mutations, and the use of several tactics to subvert the immune system of the hosts (Carocci and Yang, 2016; Guo et al., 2017; Soe et al., 2021). Given these challenges, ongoing research into therapeutic approaches for DENV infections is particularly difficult. One strategy involves repurposing existing antiviral medications to combat DENV-associated illnesses (Botta et al., 2018). To address this challenge effectively, it is crucial to employ *in silico* screening to discover potential inhibitors of DENV-2 from medicinal herbs (Rosmalena et al., 2019; Parham et al., 2020). Recently, interest in medicinal plants has rapidly increased due to their richness in phenolic compounds such as phenolic acids, flavonoids, and tannins (Alara et al., 2018; Joshi et al., 2021). The extracts of *V. cinerea* have been reported to exhibit several pharmacological properties, including antiviral activity against dengue disease (Kaushik et al., 2021). Although a large number of antimicrobial secondary metabolites have been discovered from the medicinal herb, *V. cinerea*, however, antiviral activities of these natural products have not been evaluated so far (Joshi et al., 2021; Singh et al., 2021). Computer-aided drug discovery has evolved significantly over decades, with recent years witnessing a profound integration of computational technologies in both academic research and pharmaceutical development (Rosmalena et al., 2019; Muratov et al., 2021; Sadybekov and Katritch, 2023). In this study, we computationally investigated five compounds such as Beta-amyrin, Beta-amyrin acetate, Chrysoeriol, Isoorientin, and Luteolin sourced from *V. cinerea* for their efficacy against the NSP1 of DENV-2. Our objective was to computationally evaluate the efficacy of these compounds in inhibiting NSP1 of DENV-2, providing new strategies to combat DENV infections. Nevertheless, this study highlights the need for further research into these compounds to develop more promising medications for DENV-2 associated ailments.

## 2 Materials and methods

### 2.1 Protein structure preparation

The three-dimensional (3D) crystal structure of the NSP1 of DENV-2 was obtained from the Protein Data Bank database (PDB ID: 4O6B) with a resolution of 3.00 Å. To prevent undesirable interactions and simplify the 3D structure of NSP1 by removing water and excess heteroatoms, we employed Discovery Studio Visualizer 2024 (v24.1.0.23298) (Biovia, 2015). Furthermore, we minimized the energy of NSP1 to decrease its overall potential energy, employing Swiss PDB (v4.1.0) (Wang et al., 2004). Subsequently, we identified optimal targets for conducting molecular docking studies with NSP1 of DENV-2.

### 2.2 Retrieval and preparation of compounds

Compounds from *V. cinerea* were sourced from the curated database Indian Medicinal Plants, Phytochemistry And Therapeutics 2.0 (IMPPAT 2.0) (https://cb.imsc.res.in/imppat/), which currently catalogues 4,010 Indian medicinal plants, 17,967 phytochemicals and 1,095 therapeutic uses, and related data (Mohanraj et al., 2018). Seventeen chemical structures of *V. cinerea* were retrieved from IMPPAT in pdb format. Prior to the docking analysis, each of the seventeen compounds was screened through MarvinSketch (Liliasari et al., 2021) to identify any structural discrepancies. Subsequently, the compounds were converted into PDB file format using Avogadro (v. 1.99.0) (Hanwell et al., 2012). The compounds then underwent semi-empirical geometry optimization to improve their energetic favorability. This was followed by molecular screening Gaussian (v.09) (Hasnat et al., 2024), with the protocol for geometry optimization detailed in Supplementary Material S1.

### 2.3 Molecular screening of compounds against NSP1

Molecular docking is extensively utilized in drug discovery for ligand identification. It aids in discovering potential drug candidates by predicting the binding affinity of small molecules to a target protein (Agu et al., 2023). In this investigation, we utilized PyRx (v0.8) with inbuilt AutoDock Vina to conduct molecular screening between the compounds and protein (NSP1) (Harrach and Drossel, 2014). To conduct the screening, the prepared NSP1 was designated as the macromolecule (receptor), and the compounds were designated as ligands. The docking process was executed without selecting the binding pocket’s residues. AutoDock Vina used integrated algorithms to automatically identify the active site on the protein’s surface, where substrate molecules bind and react, by analyzing the receptor’s 3D structure (Trott and Olson, 2010). This analysis involved rigid docking, where all rotatable bonds were made non-rotatable. The grid box position is crucial for effective docking analysis as it defines the specific area of the protein where ligand docking occurs (Xing et al., 2023). Considering the protein structure’s size and coverage, the grid box size was set at 67.6033 Å along the X-axis, 51.6295 Å along the Y-axis, and 56.6570 Å along the Z-axis. Among 17 compounds analyzed, five showed higher binding free energies (<−7.0 kcal/mol) and formed stable complexes with NSP1. Lacking any NSP1-specific drug compound, three general DENV-2 inhibitors were screened as controls. Compounds such as Silymarin, Baicalein, and Baicalin were known to achieve around 100% inhibition of DENV-2 (Zandi et al., 2012; Moghaddam et al., 2014; Low et al., 2021). The Discovery Studio Visualizer was utilized to visualize and measure the strength of receptor-ligand interactions.

### 2.4 Thermodynamic, molecular orbital, electrostatic potentials and spectral analyses of the phytocompounds

Compounds such as Beta-amyrin (CID 73145), Beta-amyrin acetate (CID 92156), Chrysoeriol (CID 5280666), Isoorientin (CID 114776), and Luteolin (CID 5280445) demonstrated stable interactions with NSP1, and therefore, selected for subsequent analyses along with a control drug, Baicalein (CID 5281605). These compounds and control drug underwent thermochemical property analysis using Gaussian (v.09) software to evaluate their thermodynamic properties as drug compounds (Zheng et al., 2009). To incorporate the Density Functional Theory (DFT) (Wazzan and Safi, 2017), B3LYP, and the 6-31G (d, p) basis set (Kruse et al., 2012), the gas phase geometry optimization was performed using Gaussian (v.09) software. Subsequently, the electronic transitions of the compounds were calculated using time-dependent density functional theory (TD-DFT). The highest occupied molecular orbital (HOMO) and lowest unoccupied molecular orbital (LUMO), referred to as lead molecular orbitals, were calculated using TD-DFT analysis to assess their chemical reactivity (Hossain et al., 2024). The Equations 1 and 2 (Uzzaman et al., 2021) were utilized to analyze the characteristics of molecular orbitals:

$$Gap\;\left(\Delta E\right)=\left[\varepsilon LUMO-\varepsilon HOMO\right],\eta =\frac{\left[\varepsilon LUMO-\varepsilon HOMO\right]}{2},S=\frac{1}{\eta }$$ (1)

Where: ΔE is the energy gap; *εLUMO* is the lowest occupied molecular orbit; *εHUMO* is the highest occupied molecular orbit; *η* is the chemical hardness; *S* is the chemical softness.

$$\mu =\frac{\left[\varepsilon LUMO+\varepsilon HOMO\right]}{2};\;\chi =-\frac{\left[\varepsilon LUMO+\varepsilon HOMO\right]}{2};\;\omega =\frac{\mu^{2}}{2\eta }$$ (2)

Where: *μ* is the chemical potential; *εLUMO* is the lowest occupied molecular orbit; *εHUMO* is the highest occupied molecular orbit; *χ* is the electronegativity; ω is the electrophilicity index; *μ* is the chemical potential; *η* is the chemical hardness. The obtained compounds were designated as ligands for the docking experiment.

Based on the results of DFT calculations of the five phytocompounds in Gaussian, we performed visualization of their thermodynamic, molecular orbital, electrostatic potentials, and spectral properties using GaussView (v.06) (Ofem et al., 2022). GaussView is a molecular visualization program equipped with a graphical interface that helps determine and visualize thermodynamic properties of phytocompounds (Islam et al., 2024). To elucidate the optimized thermodynamic and molecular orbital properties, we used the ResultView module of GaussView, while electrostatic potentials were measured through the Surface/Contours module. The computed simulated UV-Vis and IR spectra for optimized phytocompounds were visualized using the UV-Vis module of GaussView (Ofem et al., 2022).

### 2.5 Drug likeliness and pharmacokinetics properties analysis

In drug discovery and development, ineffectiveness, and safety are the main causes of failure. Chemical absorption, distribution, metabolism, excretion, and toxicity (ADMET) must be considered to create a better drug candidate (Lin et al., 2003). High-quality drug candidates should have satisfactory ADMET characteristics at a therapeutic dose and effective efficacy against the therapeutic target (Abdul-Hammed et al., 2021). An *in silico* pharmacokinetics method was used to analyze the time-based dynamics (ADMET) of the compounds and control drug. The SwissADME web service was used to predict the ADMET profiles of the finally optimized compounds and control drug (Daina et al., 2017). Given the importance of assessing toxicity, Protox III online server was additionally employed to verify the toxicity values (Banerjee et al., 2018). For ADMET profiling, the Simplified Molecular Input Line Entry System (SMILES) format of each compound was sourced from PubChem database (Kim et al., 2016). Furthermore, Lipinski’s Rule of Five was applied to evaluate the drug-likeness of the top five docked compounds and the control drug (Lipinski, 2004). The Pa (probability of activity) values of the corresponding compounds along with control drug was calculated using PASS Online tool of Way2Drug (Filimonov et al., 2014). The ADMET characteristics were meticulously evaluated and compared to standard ranges to determine their therapeutic potential, ensuring that each attribute falls within acceptable limits.

### 2.6 Molecular dynamics simulation

The molecular dynamics simulation (MDS) was performed for the best docked compounds and a control (Baicalein) with NSP1 protein. MDS program was conducted for a duration of 200 nanoseconds using the “Desmond v4.0 Program” of the Schrödinger software suite to model the molecular dynamics of the protein-ligand complex structures in a Linux infrastructure (El Khoury et al., 2019). Preceding to conduct MDS, receptor-ligand complexes were consolidated into a single PDB file by incorporating the textual data of the ligand file. The MDS process began with the protein preparation workflow, where the protein was optimized employing the OPLS4 force field. OPLS4 is recognized for its high accuracy and modern characteristics, offering extensive coverage of chemical space suitable for various applications in drug discovery and materials science (Lu et al., 2021). To build the environment for the molecular dynamics system, the protein-ligand complex was enveloped by a pre-defined SPC water model within an orthorhombic box (Yang and Kar, 2024). An effective MDS system was established by minimizing the system volume and automatically introducing Na^+^ ions to neutralize the system. MDS protocol was exact for all compounds where temperature and pressure were rigorously maintained at 300 K and 1.01325 bar, respectively (Hoover, 1985; Zhong and Wu, 2004). Each simulation was executed employing the constant number of particles, pressure, and temperature (NPT) ensemble, with a focus on conserving the number of atoms, pressure, and timescale (Badar et al., 2020). Throughout the MDS, long-range electrostatic interactions were computed utilizing the Particle-Mesh-Ewald method, following the approach elucidated by Essmann and Berkowitz (1999). Subsequently, the results were comprehensively analyzed and visualized using simulation interaction diagrams and MS-MD trajectory analysis techniques. Since no effective therapeutic agent has been identified against NSP1 of DENV-2, a control drug compound was not included in the MDS analysis. Therefore, to evaluate the stability of the NSP1 protein and ascertain any potential impact of the five compounds on its stability, a dedicated MDS was solely performed considering NSP1 as a reference.

### 2.7 Post MDS thermal MM-GBSA analysis

Thermal MM-GBSA (Molecular Mechanics Generalized Born Surface Area) analysis was conducted by using prime MM-GBSA, a module of the Schrödinger software suite. To run the MM-GBSA analysis, 200 ns trajectories were divided into twenty frame snapshots, poses were collected from each snapshot, and the free energy across the 200 ns simulation time was calculated. After dividing the MDS trajectory into twenty frame snapshots, the ligand and receptor were isolated for each pose to perform MM-GBSA calculations. A comprehensive analysis was conducted where 83 physicochemical properties were extracted for each ligand compound. Among these, we focused on five key properties essential for estimating the free energy of receptor-ligand interactions within biological solvent systems (Pattar et al., 2020).

## 3 Results

### 3.1 Molecular screening and interactions of the NSP1 with phytocompounds

This study focused on predicting potential drug candidates from *V. cinerea* that are effective against the NSP1 of DENV-2 using *in silico* computational approaches. Through virtual screening against NSP1, we identified five compounds (Beta-amyrin, Beta-amyrin acetate, Luteolin, Chrysoeriol, and Isoorientin) that demonstrated the strongest binding energies among the 17 constituents examined (Supplementary Table S1). Among the three control drugs (Supplementary Table S1), only Baicalein demonstrated significant binding with NSP1, with a reported binding affinity of −7.8 kcal/mol. Among the five compounds, Beta-amyrin exhibited the highest free energy of binding at −10.4 kcal/mol during docking. Similarly, the other four compounds viz. Beta-amyrin acetate, Luteolin, Chrysoeriol, and Isoorientin showed substantial binding affinities of −9.5, −7.5, −7.5, and −7.8 kcal/mol, respectively (Table 1).

**TABLE 1:** Molecular docking scores and non-bond interactions of the best five phytocompounds with NSP1.

Ligand name	Docking scores (kcal/mol)	Residue	Category	Type	Distance (Å)
Beta-amyrin (CID 73145)	−10.4	A: GLY3	H	CH	2.23882
		A: VAL5	Hp	A	4.87105
		A: ALA187	Hp	A	4.99243
		A: ALA187	Hp	A	4.77569
		A: ALA187	Hp	A	3.57247
		A: ALA187	Hp	A	4.25896
		A: LYS189	Hp	A	4.91721
		A: VAL194	Hp	A	4.83571
		A: LYS14	Hp	A	4.11929
		A: LYS189	Hp	A	4.37686
		A: ILE21	Hp	A	4.67877
		A: VAL194	Hp	A	3.73736
		A: TRP201	Hp	PA	4.88285
		A: TRP201	Hp	PA	4.79745
Beta-amyrin acetate (CID 92156)	−9.5	A: VAL5	Hp	A	5.143
		A: ALA187	Hp	A	4.31373
		A: LYS189	Hp	A	4.85946
		A: VAL194	Hp	A	4.28751
		A: VAL5	Hp	A	4.67115
		A: PHE20	Hp	PA	5.35519
Chrysoeriol (CID 5280666)	−7.5	A: LYS14	H	CH	2.8353
		A: LYS14	H	CH	2.43627
		A: SER7	H	CH	2.42767
		A: ILE19	H	CH	2.23703
		A: GLY16	H	CH	1.82358
		A: GLU12	H	CaH	3.09514
		A: ASP190	H	CaH	3.04846
		A: ASP190	H	CaH	2.55755
		A: VAL5	Hp	PA	4.84873
		A: VAL5	Hp	PA	5.35975
		A: VAL5	Hp	PA	5.07906
		A: LYS14	Hp	PA	3.73075
Isoorientin (CID 114776)	−7.8	A: GLU12	H	CH	2.30513
		A: SER7	H	CaH	2.9993
		A: CYS15	H	CaH	2.96479
		A: ASP190	H	CaH	2.31825
		A: VAL5	Hp	PA	4.02147
		A: VAL5	Hp	PA	4.92278
Luteolin (CID 5280445)	−7.5	A: GLU12	H	CH	2.29372
		A: ILE19	H	CH	2.6096
		A: GLY3	H	CH	2.78769
		A: GLY16	H	CH	1.85817
		A: GLY18	H	CaH	2.72804
		A: VAL5	Hp	PA	4.96919
		A: VAL5	Hp	PA	5.26453
		A: VAL5	Hp	PA	5.48392
		A: LYS14	Hp	PA	3.71962
Baicalein (CID 5281605)	−7.3	A: LYS14	Hp	PA	5.47362
		A: LYS14	Hp	PA	3.75664
		A: VAL5	Hp	PA	4.6627
		A: VAL5	Hp	PA	5.05427
		A: VAL5	Hp	PA	5.31521

H, Hydrogen Bond; Hp, Hydrophobic; CH, Conventional Hydrogen Bond; CaH, Carbon Hydrogen Bond; A, Alkyl; PA, Pi-Alkyl.

**FIGURE 1:**
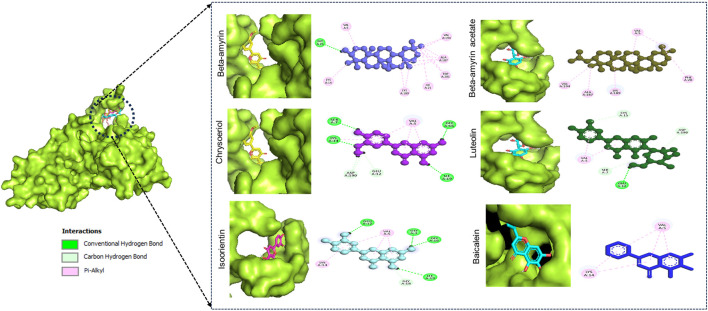
Binding affinity of the selected compounds to the identical pocket region of the NSP1 of DENV-2. Beta-amyrin showed the highest hydrophobic interactions with 13 amino acid (aa) residues compared to other four compounds. Beta-amyrin acetate showed only pi-alkyl hydrophobic interactions with five aa residues, Chrysoeriol formed three conventional hydrogen bonds and two pi-alkyl interactions, Luteolin formed four conventional H-bonds and three carbon-hydrogen bonds and a pi-alkyl interaction, and Isoorientin formed a conventional hydrogen bond, two pi-alkyl hydrophobic interactions and three carbon-hydrogen bonds.

Figure 1 illustrates the graphical representation of the interactions between these compounds and the amino acid residues of NSP1. In the initial screening, various bonded and non-bonded interactions were identified at different sites within each molecule studied. For instance, Beta-amyrin formed a conventional hydrogen bond with the GLY3 residue, with a ligand distance of 2.23882 Å. Furthermore, hydrophobic interactions were observed at 13 binding sites involving VAL5, ALA187, LYS189, VAL194, LYS14, LYS189, ILE21, and TRP201. Except for TRP201, which exhibited a pi-alkyl hydrophobic bond, all other hydrophobic interactions were of the alkyl type with the respective residues (Figure 1). The next highest free energy-releasing compound, Beta-amyrin acetate, formed alkyl bonds with VAL5, ALA187, LYS189, and VAL194 residues, and a pi-alkyl bond with PHE20. Likewise, Luteolin, formed four typical hydrogen bonds with GLU12, ILE19, GLY3, and GLY16, all within a distance of less than 3 Å. Additionally, it displayed a carbon-hydrogen bond with GLY18 and four pi-alkyl hydrophobic interactions with VAL5 and LYS14. Similarly, Chrysoeriol showed a binding affinity of −7.5 kcal/mol, forming three conventional hydrogen bonds and two pi-alkyl interactions (Figure 1). Luteolin formed four conventional hydrogen bonds with SER7, LYS14, GLY16 and ILE19, three carbon-hydrogen bonds with GLU12 and ASP190, and a pi-alkyl interaction with VAL5. Isoorientin also demonstrated one conventional hydrogen bond with GLU12, two pi-alkyl hydrophobic interactions with VAL5, and three carbon-hydrogen bonds with SER7, CYS15, and ASP190 (Figure 1; Table 1).

### 3.2 Thermodynamic properties

The five compounds with the highest negative docking scores such as Beta-amyrin, Beta-amyrin acetate, Chrysoeriol, Isoorientin, and Luteolin were further analyzed for their thermodynamic properties. All of the five compounds revealed negative free energy, indicating the spontaneous binding. The free energy values were −1,643.41 Hartree for Isoorientin and −1,400.54 Hartree for Beta-amyrin acetate, while Beta-amyrin, Chrysoeriol, and Luteolin had values of −1,247.90 Hartree, −1,068.07 Hartree, and −1,028.80 Hartree, respectively (Table 2). However, the control drug demonstrated the lowest free energy (−953.59 Hartree) compared to the selected phytocompounds. Based on these values, it can be concluded that Isoorientin and Beta-amyrin acetate exhibited greater spontaneity compared to Beta-amyrin, Chrysoeriol, and Luteolin. The enthalpy values were −1,639.32 Hartree for Isoorientin and −1,400.44 Hartree for Beta-amyrin acetate, while the enthalpy values for Beta-amyrin, Chrysoeriol, Luteolin and Baicalein were −1,247.81 Hartree, −1,068.01 Hartree, −1,028.74 Hartree, and −953.53 Hartree, respectively (Table 2). The dipole moments of Beta-amyrin, Beta-amyrin acetate, Isoorientin, Luteolin and Baicalein were determined to be 1.42 Debye, 1.77 Debye, 3.63 Debye, 5.00 Debye and 2.91 Debye, respectively. Chrysoeriol exhibited the highest dipole moment at 5.25 Debye, indicating a strong binding interaction with the receptor protein (Table 2).

**TABLE 2:** Molecular formula (MF), molecular weight (MW), energies (Hartree), and dipole moment (Debye) of compounds of *V. cinerea*.

Compound	MF	MW (g/mol)	Internal energy (Hartree)	Enthalpy (Hartree)	Gibbs free energy (Hartree)	Dipole moment (Debye)
Beta-amyrin	C30H50O	426.70	−1,247.84	−1,247.81	−1,247.90	1.42
Beta-amyrin acetate	C32H52O2	468.80	−1,400.44	−1,400.44	−1,400.54	1.77
Isoorientin	C21H20O11	448.38	−1,639.33	−1,639.32	−1,643.41	3.63
Chrysoeriol	C16H12O6	300.26	−1,068.01	−1,068.01	−1,068.07	5.25
Luteolin	C15H10O6	286.24	−1,028.75	−1,028.74	−1,028.80	5.00
Baicalein	C_15_H_10_O_5_	270.24	−953.53	−953.53	−953.59	2.91

### 3.3 Molecular orbital properties of the compounds

**FIGURE 2:**
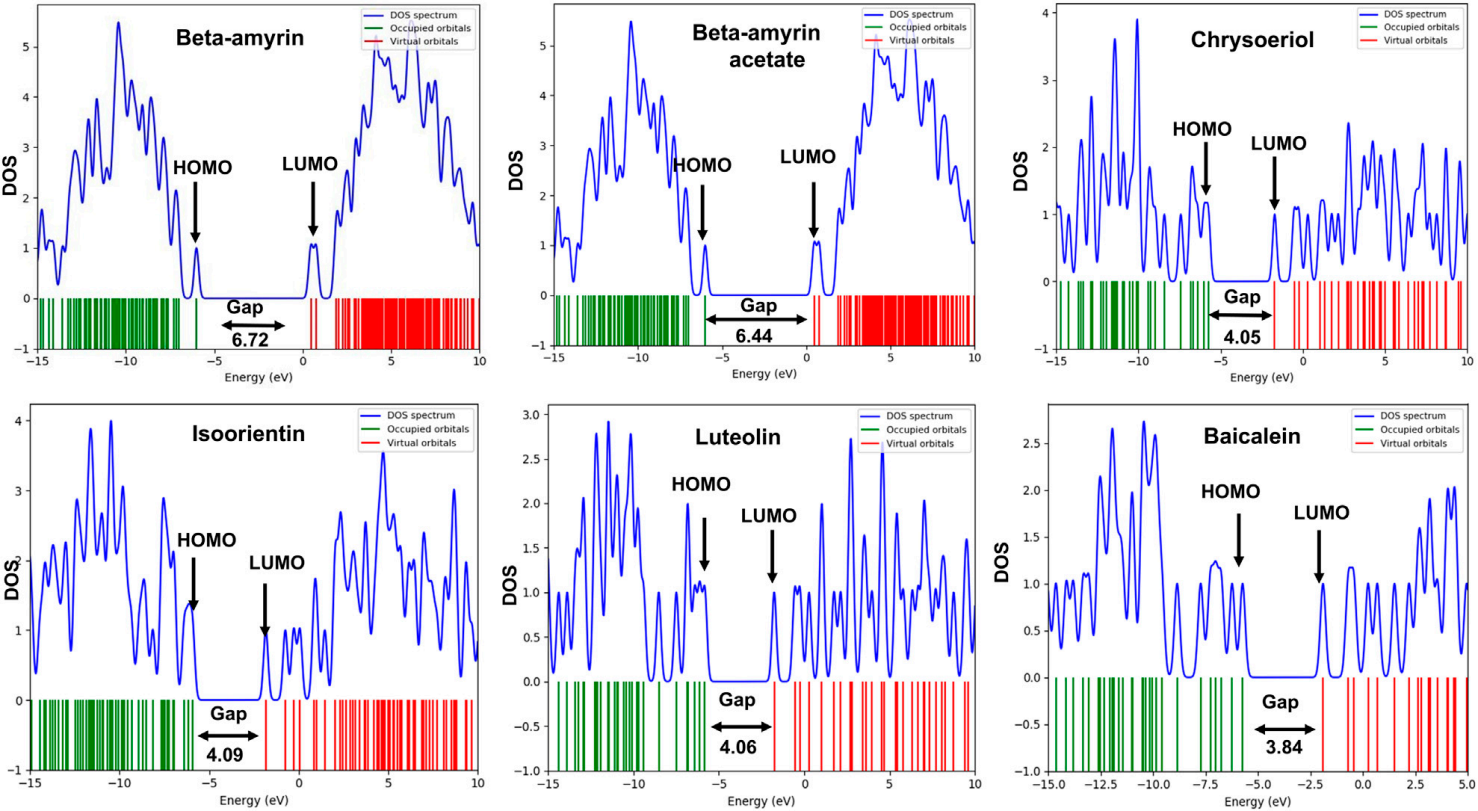
Density of state (DOS) plot of HOMO-LUMO and energy gap of five selected compounds of *V. cinerea* along with control. Green and red lines show HOMO and LUMO orbitals.

The frontier molecular orbitals (FMO), commonly referred to as the HOMO and LUMO, provided essential information on the molecular properties of the screened compounds along with the control drug. These properties included hardness (η), softness (S), chemical potential (µ), electronegativity (χ), and electrophilicity (ω), as detailed in Table 3. The energy gap (*E*_*gap*_) of the studied compounds was in the following order: Chrysoeriol < Luteolin < Isoorientin < Beta-amyrin acetate < Beta-amyrin (Table 3). The HOMO and LUMO energy gap for the control drug was found to be 3.84 which was almost close to Chrysoeriol, Luteolin and Isoorientin. The density of states (DOS) for the five chosen compounds is illustrated in Figure 2. Arrows within the figure highlighted the band gap of each molecule, color-coded to facilitate visual comparison. Chrysoeriol was identified as the softest molecule with the lowest energy gap (4.05 eV), making it highly chemically reactive but less stable. Luteolin (4.06 eV) and Isoorientin (4.09 eV) also exhibited relatively low energy gaps compared to Chrysoeriol. In contrast, Beta-amyrin had the highest energy gap (6.72 eV), followed by Beta-amyrin acetate (6.45 eV), indicating that they are the hardest molecules with lower reactivity but greater stability (Figure 2; Table 3).

**TABLE 3:** Energy (eV) of HOMO-LUMO, gap, hardness (η), softness (S), chemical potential (µ), electronegativity (χ), and electrophilicity (ω) of the compounds of *V. cinerea*.

Compound	ɛHOMO	ɛLUMO	Energy gap (*E*_*gap*_)	η	S	µ	χ	Ω
Beta-amyrin	−5.98	0.73	6.72	3.36	0.15	−2.63	−3.36	1.03
Beta-amyrin acetate	−5.98	0.46	6.45	3.23	0.15	−2.77	2.77	1.19
Isoorientin	−5.92	−1.83	4.09	2.04	0.24	−3.87	−0.24	3.74
Chrysoeriol	−5.74	−1.68	4.05	2.02	0.25	−3.71	−2.02	3.40
Luteolin	−5.82	−1.76	4.06	2.03	0.49	−3.79	3.79	3.54
Baicalein	−5.74	−1.90	3.84	1.92	0.52	−3.82	3.82	3.79

### 3.4 Molecular electrostatic potentials of the compounds

**FIGURE 3:**
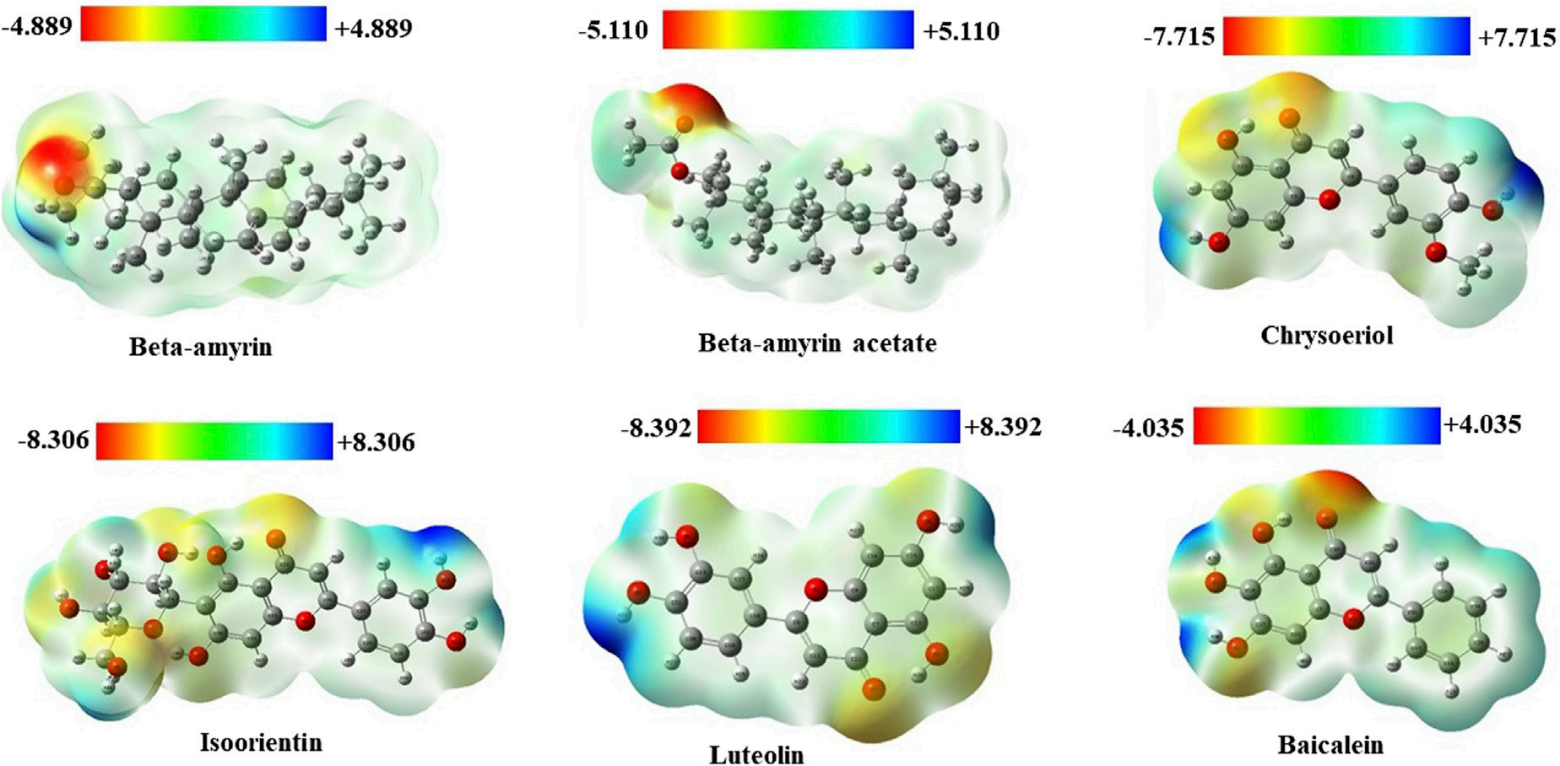
Molecular electrostatic potential (MEP) map of five selected compounds of *V. cinerea* along with control. Different colors indicate charge distribution such as blue for positive charge, red for negative charge, and green for neutral charge. Short electron density and poor interaction are identified in the blue region, while the red region indicates high electron density and potential interaction.

Molecular electrostatic potential (MEP) analysis aided in understanding H-bonding interactions and the biological recognition process of the selected compounds. In this study, we found the highest positive potential at the most electropositive atom, hydrogen, and the highest negative potential at the most electronegative atom, oxygen. In the MEP analysis, Luteolin showed the highest positive potential at +8.392 atomic units (a.u.) and the most negative potential at −8.392 a.u. The other four compounds, namely, Beta-amyrin, Beta-amyrin acetate, Chrysoeriol, and Isoorientin exhibited negative potential values of −4.889, −5.110, −7.715, and −8.306 a.u., respectively, and positive potential values of +4.889, +5.110, +7.715, and +8.306 a.u., respectively (Figure 3). Additionally, the distribution of the MEP for the control drug ranged from −4.035 to +4.035 a.u. The MEP analysis facilitated the identification of reactive sites by distinguishing electrophilic and nucleophilic regions within both the five compounds and the control drug. This was achieved through color gradients that represented positive, negative, or zero electrostatic potentials (Figure 3).

### 3.5 Infrared spectroscopy of the compounds

**FIGURE 4:**
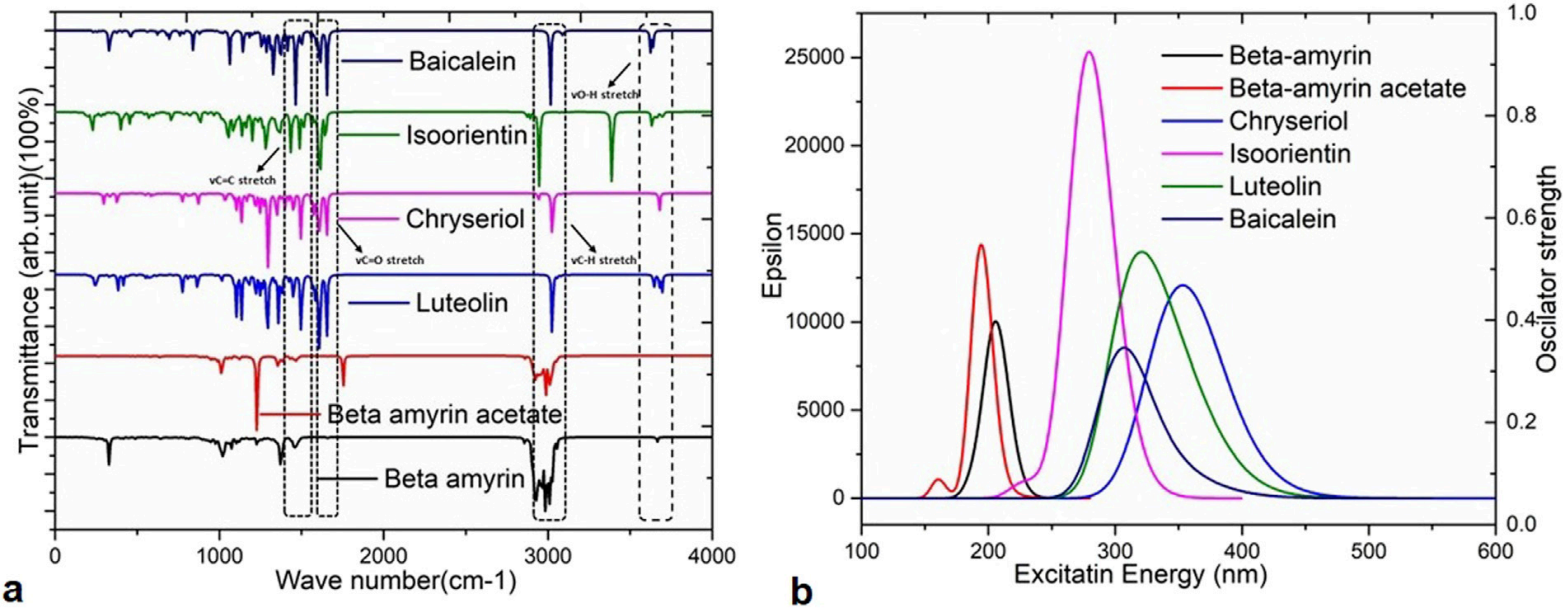
Vibrational frequencies and UV-Visible spectra of the selected five compounds of *V. cinerea* and the control drug. **(A)** Values of vibrational frequency (C = O, O–H, C = C and C–H stretches) are indicated by different arrows. **(B)** The UV-Visible spectra display the maximum absorbance (epsilon; λ_max_) values, excitation energies, and oscillator strengths of the selected compounds. Peaks of different colors correspond to specific compounds.

Infrared (IR) spectroscopy or Fourier transform infrared (FTIR) spectroscopy is utilized to analyze unknown substances by studying their molecular vibrations. The IR region spans the frequency range of the spectrum from 12,500 to 10 cm^−1^. In this analysis, we used the frequency between 0–4,000 cm^−1^ for five compounds such as- Beta-amyrin, Beta-amyrin acetate, Isoorientin, Chrysoeriol and Luteolin (Figure 4A). The vibrational frequency values were scaled by a factor of 0.9627 to ensure their accuracy aligns with experimental data compared to standard sources. The C-H^a^ stretching was observed at 3,008 cm^−1^ for Beta-amyrin, 2,940 cm^−1^ for Beta-amyrin acetate, 3,124 cm^−1^ for Isoorientin, 3,130 cm^−1^ for Chrysoeriol, and 3,122 to 3,026 cm^−1^ for Luteolin. The C-H vibrational frequency bands were at 3,025 cm^−1^ for Beta-amyrin, 20,952 cm^−1^ for Beta-amyrin acetate, and 3,032 cm^−1^ for Chrysoeriol, aligned with the experimental vibrational range. Beta-amyrin and Chrysoeriol also showed C = C stretching at 1,661 and 1,599 cm^−1^ vibrational frequencies, respectively. These results were significantly similar to the experimental results (Figure 4A; Supplementary Table S2). C = C^a^ stretching was found at 1,662 cm^−1^ for Beta-amyrin acetate and 1,560 cm^−1^ for Isoorientin. Beta-amyrin acetate and Chrysoeriol did not show O-H stretching in their spectra. In contrast, Beta-amyrin, Isoorientin, and Luteolin exhibited O-H stretching at frequencies of 1,739, 3,697 cm^−1^, and 3,695 to 3,645 cm^−1^, respectively, falling within the experimental value ranges. Beta-amyrin acetate and Chrysoeriol exhibited notable C=O stretching at frequencies of 1,754 and 1,589 cm^−1^, respectively, aligning closely with the experimental values of 1,822.47 and 1,651 cm^−1^. In contrast, Isoorientin and Luteolin showed C=O stretching at frequencies of 1,646 and 1,654 cm^−1^, respectively. Compared to the studied compounds, the experimental and scaled vibrational frequency values of the control drug, Baicalein, were found to be similar, with only a few discrepancies (Figure 4A; Supplementary Table S2).

### 3.6 UV-visible spectral properties of the compounds

In this study, time-dependent density functional theory (TD-DFT) calculations using the B3LYP/631G (d,p) method were performed for each compound to elucidate the electronic transitions and spectra within the molecules. The energy states, wavelengths (nm), excitation energies, transition configurations, and oscillator strengths for each compound are presented in Supplementary Table S3 and the UV-visible spectra are illustrated in Figure 4B. In this investigation, absorption wavelengths were observed between 0 and 1,000 nm in the UV-vis region. The molecules showed maximum absorption wavelengths (λ_max_) corresponding to electron charge transfer from S_0_→S_1_ states, specifically 357.70 nm (λ_max_ = 0.6665 H→L) for Chrysoeriol and 352.26 nm (λ_max_ = 0.65851 H→L) for Luteolin.

Their respective oscillator strengths were 0.2422 f and 0.1293 f. Both compounds exhibited low excitation energies of 3.466 and 3.5197 eV, respectively, indicating increased potential for chemical reactivity due to reduced HOMO-LUMO gaps (Supplementary Table S3). Isoorientin exhibited λ_max_ at 237.31 nm with a low excitation energy of 4.4360 eV and an oscillator strength of 0.6252 f. In contrast, Beta-amyrin showed the highest excitation energy (6.010 eV), with a wavelength of 206.27 nm and an oscillator strength of 0.2402 f (Figure 4B). Therefore, it can be inferred that Beta-amyrin demonstrated lower chemical reactivity compared to other compounds studied in this research. However, the control drug, Baicalein, showed a λ_max_ at 354.51 nm with a low excitation energy of 4.0297 eV and an oscillator strength of 0.0993 f, closely resembling the values for Chrysoeriol and Luteolin (Figure 4B; Supplementary Table S3).

### 3.7 Drug likeness, pharmacokinetic and toxicological properties of the compounds

The drug-likeness and absorption, distribution, metabolism, and excretion (ADME) profiles of the five compounds extracted from *V. cinerea* are detailed in Table 4. Each compound adhered to Lipinski’s “rule of 5”, with a molecular weight below 500 Da. Among them, Beta-amyrin and Beta-amyrin acetate demonstrated strong lipid solubility (4.74 and 5.19 Log P, respectively), while Chrysoeriol showed moderate solubility with a score of 2.44 Log P. In contrast, Luteolin and Isoorientin exhibited lipid insolubility with Log P values of 1.86 and 2.12, respectively, in the ADME structure-activity relationship. Among these compounds, Chrysoeriol and Luteolin demonstrated significant gastrointestinal (GI) absorption. Additionally, the compounds showed varying degrees of synthetic accessibility: Beta-amyrin (6.04), Beta-amyrin acetate (5.98), Chrysoeriol (3.06), Isoorientin (5.04), Luteolin (3.02) and Baicalein (3.02). The molar refractivity of all compounds fell within the specified range outlined in Lipinski’s criteria. The Pa values of the screened compound were consistently higher than those of the control drug (Table 4).

**TABLE 4:** Drug likeness and pharmacokinetic (ADMET) properties of the compounds of *V. cinerea*.

Name	M. W. (g/mol)	Heavy atoms	A. Heavy atom	Rotatable bond	H-bond acceptors	H-bond donors	Log P (iLogP)	Logs (ESOL)	Pa value	GI absorption	Lipinski	Synthetic accessibility	Molar refractivity
Beta -amyrin	426.72	31	0	0	1	1	4.74	Poor	0.81	Low	Yes	6.04	134.88
Beta-amyrin acetate	468.75	34	0	2	2	0	5.19	Poorly	0.85	Low	Yes	5.98	144.62
Chrysoeriol	300.26	22	16	2	6	3	2.44	Moderate	0.75	High	Yes	3.06	80.48
Isoorientin	448.38	32	16	3	11	8	2.12	Soluble	0.48	Low	No	5.04	108.63
Luteolin	286.24	21	16	1	6	4	1.86	Soluble	0.41	High	Yes	3.02	76.01
Baicalein	270.24	20	16	1	5	3	2.43	Moderate	0.39	High	No	3.02	73.99

To assess the toxicity of the five selected compounds, we employed Protox III and AdmetSAR as *in silico* toxicity testing tools. The analysis revealed that each compound was inactive regarding hepatotoxicity and cytotoxicity. However, the results for mutagenicity, immunotoxicity, and carcinogenicity varied among the compounds (Supplementary Table S4). For instance, Beta-amyrin, Beta-amyrin acetate, and Chrysoeriol were found to be non-mutagenic. Conversely, Isoorientin and Luteolin exhibited potential mutagenic properties. Based on immunotoxicity data, Beta-amyrin and Beta-amyrin acetate were identified as immunotoxic, while Isoorientin, Luteolin, and Chrysoeriol were found to be non-immunotoxic. Furthermore, Beta-amyrin acetate and Luteolin were classified as carcinogens, whereas Beta-amyrin, Chrysoeriol, and Isoorientin showed no carcinogenic activity.

According to the acute oral toxicity (AOT) results, all the compounds were classified as Category III, indicating their suitability for oral administration. On the other hand, the control drug showed a mixed toxicity profile in both Protox III and AdmetSAR analyses (Supplementary Table S4).

### 3.8 Molecular dynamics of the NSP1 and phytocompounds

**FIGURE 5:**
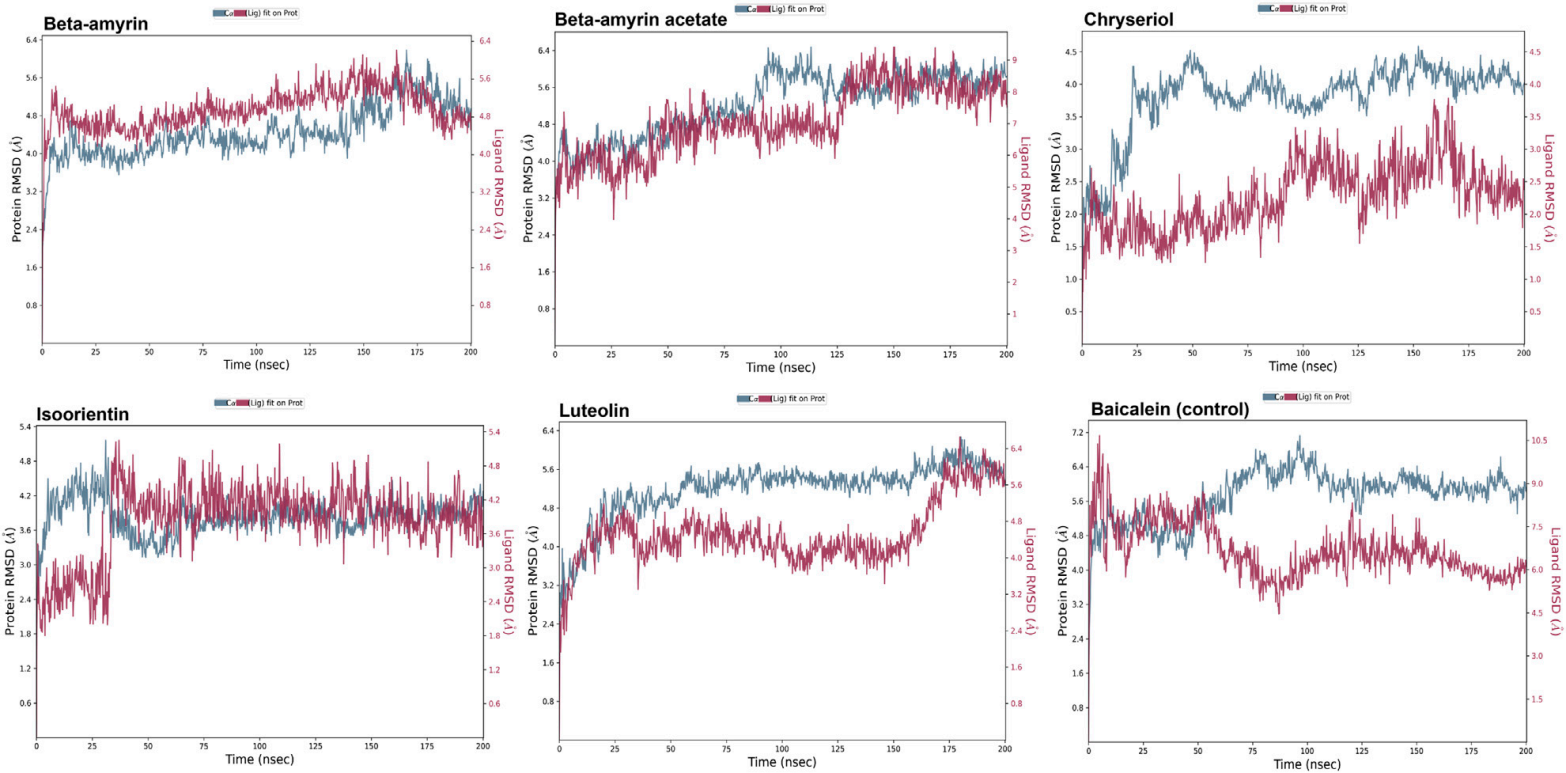
RMSD value of the studied compounds demonstrated the overall stability of protein-ligand complex throughout the 200 ns trajectory.

**FIGURE 6:**
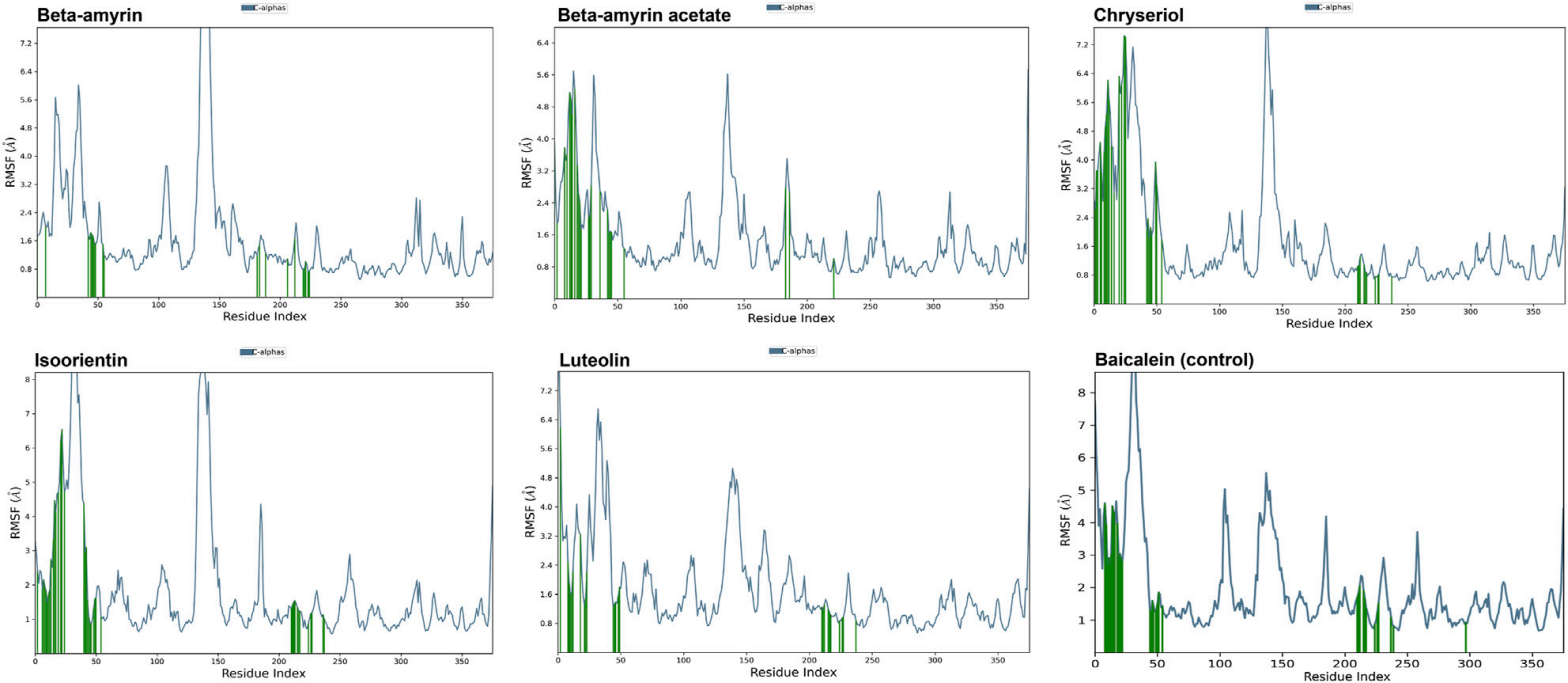
RMSF value of the studied compounds demonstrate the protein stability throughout the 200 ns trajectory.

A molecular dynamics simulation (MDS) was conducted over 200 ns to assess the stability and strength of interactions between NSP1 and the five selected compounds along with control drug Baicalein. As depicted in Figure 5, Beta-amyrin, Isoorientin, and Chrysoeriol exhibited fluctuations ranging from 1 to 5 Å. In contrast, Beta-amyrin acetate and Luteolin and Baicalein displayed broader fluctuations, ranging from 1 to 9 Å. One of the key findings of this study is that none of the compounds exhibited fluctuations from the binding pocket regions of NSP1 throughout the 200 ns trajectories except control (Supplementary Material S2). However, Beta-amyrin acetate and Luteolin exhibited higher RMSD values (Figure 5), suggesting fluctuations even though the binding to NSP1 pocket regions did not alter the pocket conformation. To understand the reasons behind these higher RMSD values, we analyzed the RMSF curves. The RMSF curves of the five compounds showed variations, but notably, the regions (150–250) where the compounds strongly bound exhibited fluctuations lower than 2 Å (Figure 6 and Supplementary Material S2). From the 200 ns RMSF trajectories, it was evident that certain other parts of the protein chain had higher fluctuations, affecting the overall RMSD. To obtain more accurate insights into the elevated RMSD and RMSF values, we conducted a control molecular dynamics simulation for NSP1 without any ligand compounds and a control compound (Baicalein). This control simulation revealed that the NSP1 exhibited higher RMSF values on its own, suggesting inherent instability in certain regions of the crystallographic protein. Additionally, these regions are implicated in the observed increase in RMSD values (Supplementary Figure S1). The RMSF curve for Baicalein exhibited similar fluctuations to the control compound, although the control compound showed greater fluctuation at the beginning of the curve (Figure 6). The overall RMSD of NSP1 fluctuated up to 6 Å. However, the binding of Chrysoeriol and Isoorientin with NSP1 demonstrated lower RMSD values, indicating that the complexes formed with these compounds were more stable than the unbound form of NSP1.

**FIGURE 7:**
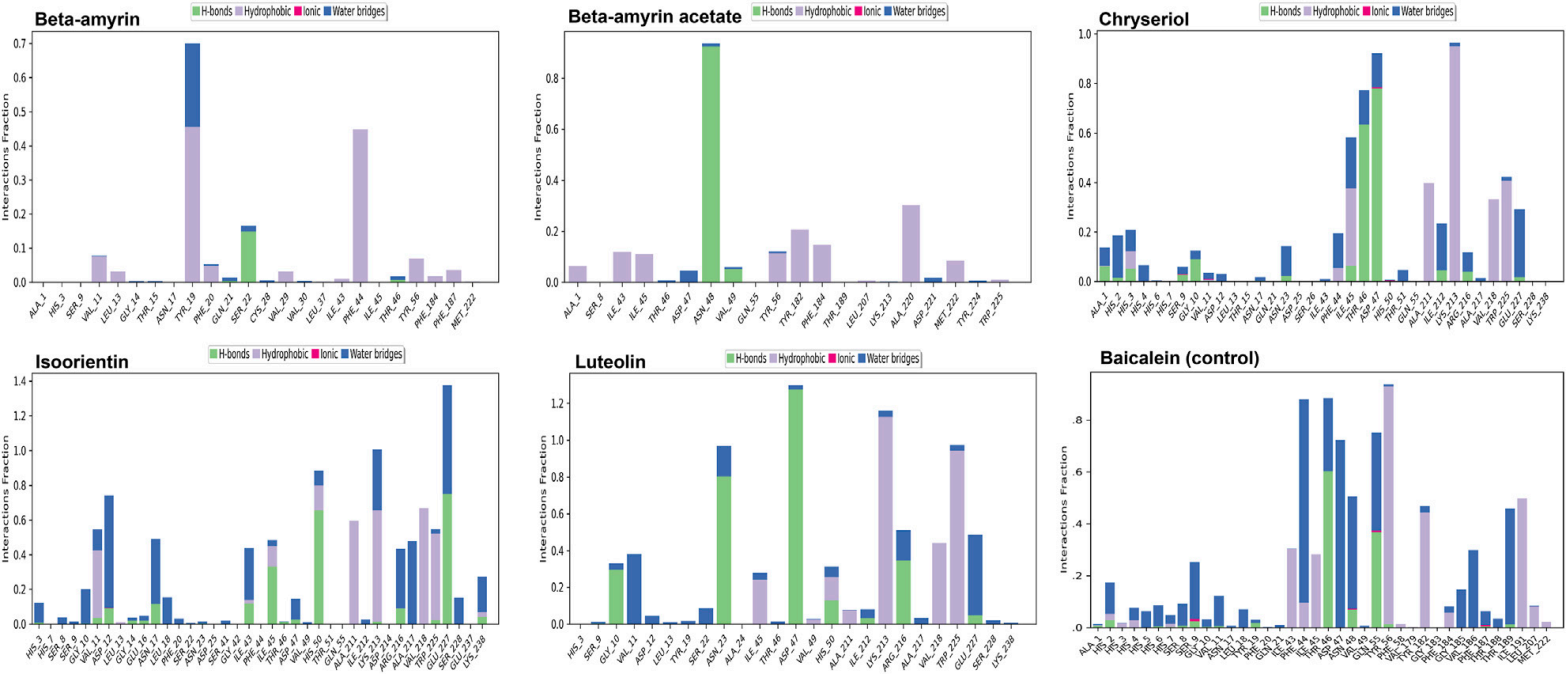
The interaction fraction indicates that these residues involved in protein-ligand contacts strongly.

**FIGURE 8:**
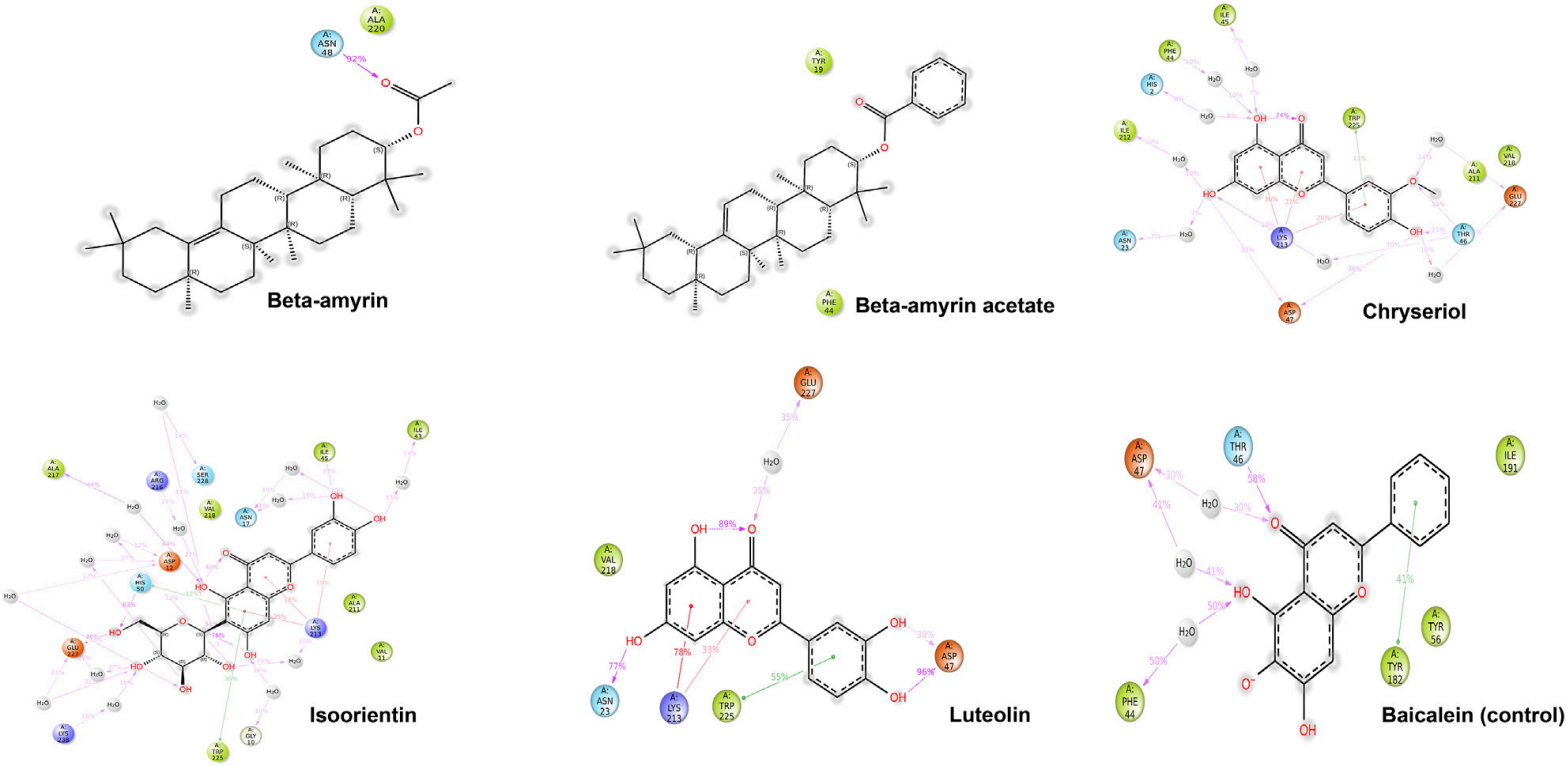
The detailed view of both covalent and non-covalent interactions between the receptor and the ligands over the 200 ns trajectories.

Compounds Beta-amyrin and Beta-amyrin acetate exhibited strong non-covalent interactions with NSP1. In contrast, the other three compounds, Luteolin, Chrysoeriol, and Isoorientin, displayed strong covalent interactions with the NSP1 binding pockets amino acids that fall in the 150–250 regions (Figures 7, 8). The MDS results indicated that Luteolin, Chrysoeriol, and Isoorientin could inhibit the function of NSP1 of DENV-2, as they demonstrated strong covalent interactions throughout the trajectory periods (Supplementary Material S2). Comparison of the dynamics trajectories of the five compounds and the control revealed that Luteolin, Chrysoeriol, and Isoorientin outperformed the control in all MDS parameters. While the control compound, Baicalein, remained bound to the same binding pocket as the other studied compounds, it showed significant displacement in the pocket regions as the simulation progressed. In contrast, none of our studied compounds exhibited such displacement during the simulation period (Supplementary Material S2). The higher RMSD fluctuations of up to 10 Å for the control further confirm that the five studied compounds performed better than the control (Figure 5). To gain a deeper understanding of these five compounds, the dual method (post-MDS MM-GBSA) was applied to confirm the efficiency of binding and stable interactions between the ligands and target proteins.

### 3.9 Post MDS thermal MM-GBSA

**FIGURE 9:**
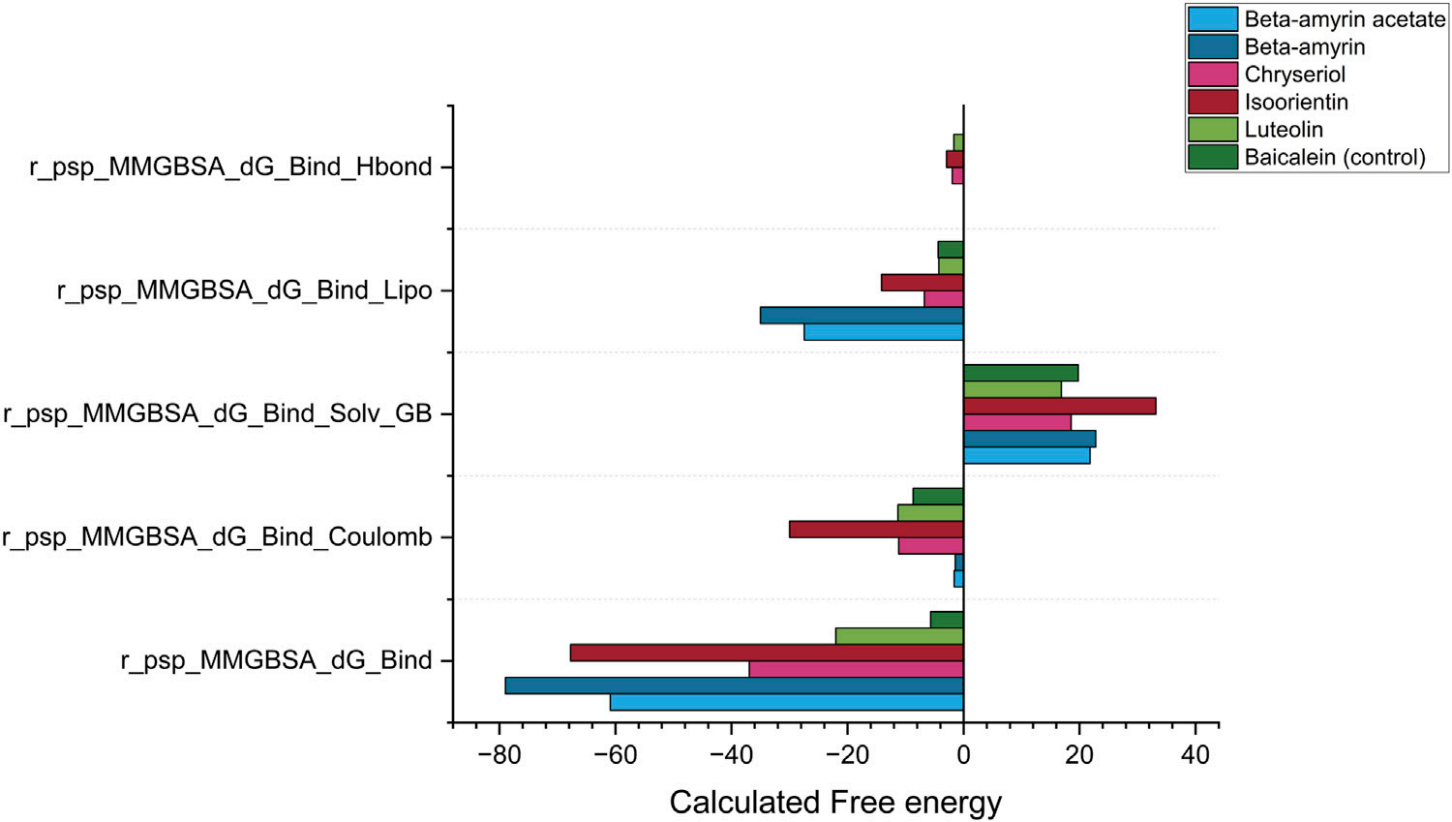
Post simulation thermal MM-GBSA analysis for NSP1, five phytocompounds of *V. cinerea* and control drug (Baicalein). The total MM-GBSA binding free energy was calculated, considering the Gibbs free energy, electrostatic energy (coulombic), generalized born electrostatic solvation energy, hydrogen bonding (H-bond) and lipophilic effects during binding to the receptor NSP1.

The MM-GBSA method was employed to estimate the free energies released from the interaction between NSP1 of DENV-2 and the compounds of *V. cinerea*. The most critical parameter, Gibbs free energy during binding (r_psp_MMGBSA_dG_Bind), demonstrated a negative value for each ligand-protein complex (Figure 9). A negative free energy indicates that the formation of the complex releases more energy than it consumes, signifying that the interaction between the compounds and NSP1 occurred spontaneously. Compared to Baicalein, all five compounds demonstrated stronger spontaneous interactions with NSP1. The next critical parameter was the release of electrostatic energy upon binding (r_psp_MMGBSA_dG_Bind Coulomb). In this parameter, Isoorientin released the highest free electrostatic energy upon binding with NSP1, revealing direct electrostatic interactions between the compounds in the bound state *versus* the unbound state. This parameter supports the MDS results, where Luteolin, Chrysoeriol, and Isoorientin exhibited better electrostatic interactions than both Beta-amyrin and the control.

The next key parameter was the generalized Born electrostatic solvation energy (Bind Solv GB). The solvent molecules had a positive effect on the energetics of both the control and all studied compounds. A positive value for electrostatic solvation energy indicated that these compounds did not readily dissolve in water. Finally, two other critical parameters, the hydrogen bonding (H-bond) and lipophilic effects on the binding free energy of the system, were calculated as negative (Figure 9). Comparing all five parameters described the reasons for the suitable covalent and non-covalent interactions with NSP1. The energetically favorable interactions illustrated that these compounds can disrupt the biological function of NSP1.

## 4 Discussion

The discovery and development of new drugs are both costly and time-consuming. A strategic computational approach to rational drug design has demonstrated its value by assessing the potential of selected phytochemicals before wet-lab experiments. This approach predicts their pharmacodynamic and pharmacokinetic properties, such as electrostatic potential, drug-likeness, oral bioavailability, efficacy, toxicity risk, and other relevant characteristics. In-silico screening and prediction of compounds with favourable properties significantly reduce research costs and time. This study identified potential inhibitors in *V. cinerea* for NSP1, a key virulence factor of DENV-2. Extracts from *V. cinerea*, a plant traditionally used for its medicinal properties (Alara et al., 2018; Joshi et al., 2021; Singh et al., 2021), were examined for their antiviral potential. We evaluated the inhibitory potential of five selected compounds such as Beta-amyrin, Beta-amyrin acetate, Chrysoeriol, Isoorientin, and Luteolin which demonstrated strong interactions with NSP1 of DENV-2 in virtual screenings, suggesting significant antiviral properties.

To compute the drug potential of the compounds under study, all active phytoconstituents were tested through thermodynamic calculations. This method allowed us to anticipate the reaction kinetics and chemical stability of the selected compounds. In this study, all calculated energies were found to be negative, which indicated the spontaneous binding potency of selected compounds with NSP1 and their high values suggest more available binding (Azam et al., 2018; Uddin et al., 2020). The values of Gibbs free energy, enthalpy, and internal energy of a compound are used to predict the spontaneity of a reaction when interacting with a receptor (Uzzaman and Uddin, 2019). The compounds underwent further evaluation using FMO analysis to assess their chemical reactivity and kinetic stability. This analysis focused on the energy difference between the HOMO-LUMO (Figure 2), providing insights into the potential interactions and stability of the compounds in biological systems (Fernández and Bickelhaupt, 2014). Large HOMO-LUMO gaps indicated minimal chemical reactivity due to high kinetic stability and *vice versa* (Aihara, 1999). Based on the FMO analysis, Chrysoeriol, Luteolin, and Isoorientin were expected to exhibit higher chemical reactivity compared to Beta-amyrin and Beta-amyrin acetate. Another crucial parameter studied was the electrostatic potential, which identified reactive sites in the compounds whether they were electrophilic or nucleophilic. This analysis demonstrated the size and shape of molecules with positive, negative, or zero potential (Figure 3). Furthermore, it facilitated the understanding of the chemical reactivity of the compounds in the biological recognition process (Politzer and Murray, 1991). The IR spectroscopy analysis of the compounds revealed that the spectra of all five compounds fell within the experimental value range for the IR region, spanning frequencies from 12,500 to 10 cm⁻^1^ (Figure 4). Moreover, UV-Vis spectroscopy analysis of the selected compounds revealed that Chrysoeriol, Luteolin, and Isoorientin exhibited maximum absorption wavelengths associated with lower excitation energies, indicating a higher chemical reactivity compared to Beta-amyrin and Beta-amyrin acetate. UV-Vis spectroscopy analysis of compounds is routinely employed to investigate electronic energy levels, providing insights into the characteristics of conjugated multiple bonds and aromatic rings (Yadav, 2005). However, during the docking analysis, Beta-amyrin and Beta-amyrin acetate demonstrated the highest binding free energy compared to Chrysoeriol, Luteolin, and Isoorientin. Molecular docking helps to simulate and predict the interaction between small molecules (such as drugs or compounds) and larger biomolecules (such as proteins or nucleic acids) in order to understand their binding affinity, interaction mode, and potential biological activity (Ballester and Mitchell, 2010). More negative binding free energies indicate stronger interactions between the ligand and the receptor protein (Gilson and Zhou, 2007). However, Beta-amyrin and Beta-amyrin acetate actually exhibited low binding free energies, as lower (more negative) binding free energy corresponds to higher binding affinity, whereas higher binding free energy indicates lower binding affinity (Figure 1; Table 1). Besides molecular docking, factors like hydrogen bonding, hydrophobic interactions, and optimal protein-ligand distance play crucial roles in ligand binding efficacy and stability (Patil et al., 2010). Covalent pairings between receptors and ligands enhance affinity by reducing water molecule interference, while strong hydrogen bonds further enhance binding affinity (Chen et al., 2016).

Through chemical property and docking analyses, all five compounds demonstrated potency against NSP1. In the chemical property analysis, Chrysoeriol, Luteolin, and Isoorientin showed better reactivity, while in docking analysis, Beta-amyrin and Beta-amyrin acetate exhibited better binding. For final validation of their inhibitory activities against NSP1, we employed MDS, evaluating drug–target interactions over time to accurately predict ligand-receptor binding practicalities (Figure 5). MDS provided detailed insights into drug-target interactions over time, enabling accurate predictions of ligand-receptor binding specificity. During the simulations, Chrysoeriol and Isoorientin exhibited the most stable interactions, indicated by the lowest RMSD fractions. RMSD curves showed protein conformation fluctuations, with values reaching up to 9 Å. Despite this, interactions between the compounds and NSP1 remained uninterrupted (Supplementary Material S2).

Previous studies suggest high RMSD values indicate poor interactions, yet none of the compounds disengaged (Wu et al., 2020; Wu et al., 2024). NSP1 also adopted more energetically favorable conformations when interacting with these compounds. In MDS, the five studied compounds showed stronger inhibitory effects against NSP1 than control drug Baicalein, an effective drug against DENV-2 (Zandi et al., 2012; Moghaddam et al., 2014; Low et al., 2021). Our studied compounds, similar to the control drug, exhibited binding to the pocket residues through covalent and non-covalent interactions, maintaining stability at this position throughout the 200 ns simulation period (Supplementary Material S2). The higher RMSD values observed for the control drug indicate significant displacement from the binding pocket (Kakhar Umar et al., 2023). Notably, this displacement was only reported for the control, reinforcing the superior binding stability and effectiveness of the five studied compounds (Supplementary Material S2). Although the control drug Baicalein displayed the lowest HOMO-LUMO energy gap compared to the selected five compounds from *V. cinerea*, indicating potentially high chemical reactivity (Figure 2; Table 4), further evaluation of their interactions with NSP1 of DENV-2 revealed that Chrysoeriol, Luteolin, and Isoorientin demonstrated more favorable binding affinities. This suggests that, despite Baicalein’s advantageous electronic properties, the selected phytocompounds exhibited stronger and potentially more effective interactions with the target protein, making them promising candidates for further development as inhibitors of NSP1 in DENV-2. Overall, the five studied compounds exhibited potential inhibitory activities against NSP1 (Figures 5–8, Supplementary Material S2). Lipinski’s “rule of 5” assessed strong absorption and permeation for compounds with a molecular weight under 500, a log P of 5.0 or lower, no more than five hydrogen bond donors, and no more than ten hydrogen bond acceptors. Failure to adhere to these criteria could compromise the compound’s ability to be effectively absorbed and distributed in the body, impacting its overall bioavailability and therapeutic efficacy (Shaikh et al., 2020; Tabassum and Ahmad, 2020). Assessing the toxicity potential and bioavailability of compounds was essential as it enabled us to gauge the safety and effectiveness of these substances as potential therapeutic agents. Among the five compounds assessed, only Chrysoeriol and Luteolin showed significant bioavailability, GI absorption, lipophilicity, and accessibility (Table 4), suggesting efficient absorption and transport kinetics. Moreover, in the toxicity risk assessment, only Chrysoeriol met all the safety parameters. Overall, Chrysoeriol performed well in both thermodynamic and pharmacokinetic studies and emerged as a potential inhibitor based on our investigation. Given the absence of a drug targeting NSP1 of DENV-2, the chosen compounds require additional evaluation for their drug-like characteristics, followed by *in-vitro* and *in-vivo* investigations to screen for potential future drugs.

## 5 Conclusion

DENV-2 is one of the four types of DENVs that cause dengue fever. Currently, there is no specific antiviral treatment available for DENV-2 infections. The findings from this study represent the first evidence suggesting that compounds of *V. cinerea* could serve as an alternative source for discovering novel inhibitors against the NSP1 of the DENV-2 through computer-aided screening. We evaluated 17 compounds from *V. cinerea* for their effectiveness, primarily targeting NSP1 of DENV-2. Through a variety of screening tests, we identified five compounds, namely, Beta-amyrin, Beta-amyrin acetate, Isoorientin, Luteolin, and Chrysoeriol that effectively inhibited the NSP1. The results indicated that the screened compounds exhibited substantial binding affinities, suggesting potential as NSP1 inhibitors. The selected compounds of *V. cinerea* adhered to Lipinski’s “rule of 5”, with varying lipid solubility and GI absorption. Toxicity analysis showed no hepatotoxicity and cytotoxicity, with mixed mutagenicity, immunotoxicity, and carcinogenicity profiles. In a 200 ns MDS, compounds Beta-amyrin, Isoorientin, and Chrysoeriol showed stable fluctuations (1–5 Å), while Beta-amyrin acetate and Luteolin exhibited broader fluctuations (1–9 Å) without altering NSP1 binding pockets. Additionally, MM-GBSA analysis revealed that the interaction of compounds with NSP1 occurred spontaneously and significantly disrupted NSP1 function. Overall, only Chrysoeriol demonstrated favorable drug likeliness, emerging as a potential inhibitor of NSP1, offering new avenues for the treatment and management of DENV-2 infections. Further *in-vitro* and *in-vivo* studies are essential to validate its potential as a future therapeutic agent.

## Data Availability

The datasets presented in this study can be found in online repositories. The names of the repository/repositories and accession number(s) can be found in the article/Supplementary Material.
