# Supplementary material for: Computational identification of Vernonia cinerea-derived phytochemicals as potential inhibitors of nonstructural protein 1 (NSP1) in dengue virus serotype-2
Source: Front Pharmacol. 2024 Oct 15;15:1465827. doi: 10.3389/fphar.2024.1465827 (PMC11518830; doi:10.3389/fphar.2024.1465827)
Supplement: Supplementary file 1 [file DataSheet1.zip › Supplementary file 2.PDF]

File S2. Please find all the animation movies of the drug candidates and control drug through---

https://doi.org/10.6084/m9.figshare.26892421.v2 (Title: **Computational Identification of Vernonia cinerea-Derived Phytochemicals as Potential Inhibitors of Nonstructural Protein 1 (NSP1) in Dengue Virus Serotype-2)**
