# Supplementary material for: Computational identification of Vernonia cinerea-derived phytochemicals as potential inhibitors of nonstructural protein 1 (NSP1) in dengue virus serotype-2
Source: Front Pharmacol. 2024 Oct 15;15:1465827. doi: 10.3389/fphar.2024.1465827 (PMC11518830; doi:10.3389/fphar.2024.1465827)
Supplement: Supplementary file 1 [file DataSheet1.zip › Supplementary figures and tables.PDF]

Supplementary Tables

Table S1: Docking scores of selected phytocompounds from *Vernonia cinerea* against NSP1 of DENV-2 and control drugs.

Serial No.	Compounds Name	Docking Score (kcal/mol)
1	Beta amyrin	-10.4
2	Beta-amyrin acetate	-9.5
3	Isoorientin	-7.8
4	Chrysoeriol	-7.5
5	Luteolin	-7.5
6	Alpha amyrin	-7.1
7	Delta -amyrin acetate	6.9
8	Beta-amyrin benzoate	6.5
9	Lupeol	-6.1
10	Lupeol acetate	-6.0
11	Alpha-amyrin acetate	-5.8
12	Spinasterol	-7.8
13	Cynaroside	-6.3
14	Sitosterol	-7.0
15	Stigmasterol	-6.6
16	Campesterol	-4.6
17	Oleic acid	-4.1
18	Baicalein (Control)	-7.8
19	Silymarin (Control)	-3.6
20	Baicalin (Control)	-4.1

Table S2: Selected vibrational frequencies (cm^{-1}) *Veronica Cinerea* of calculated in gas phase (scaled).

Name	Assignments	Vibrational Frequencies (cm-1) (experimental)	Vibrational Frequencies (cm-1) (Scaled)
Beta-amyrin	νC-H[a] Strech<br>νC-H Stretch<br>νC=C Stretch<br>νO-H Strech	3125<br>3143<br>1726<br>1807	3008<br>3025<br>1661<br>1739
Beta-amyrin acetate	νC-H Stretch<br>νC-H[a] Strech<br>νC=O Stretch<br>νC=C[a] Stretch	3066.22<br>3054.39<br>1822.47<br>1726.47	2952<br>2940<br>1754<br>1662
Isoorientin	νO-H stretch<br>νC-H[a] stretch<br>νC=O[a] stretch<br>νC=C [a] stretch	3841<br>3246<br>1710<br>1621	3697<br>3124<br>1646<br>1560
Luteolin	νO-H stretch<br>νC-H[a] stretch<br>νC=O[a] stretch<br>νC-C[a] stretch	3837.95-3786.65<br>3243-3143.30<br>1718.02<br>1677.45	3695-3645<br>3122-3026<br>1654<br>1615
Chrysoeriol	νC-H[a] Stretch<br>νC-H Stretch<br>νC=O Stretch<br>νC=C Stretch	3251<br>3150<br>1651<br>1661	3130<br>3032<br>1589<br>1599
Baicalein (Control)	νC-Ha Strech<br>νC=O Stretch<br>νC=C Stretch<br>νO-H Strech	3248<br>1675<br>1537<br>3782	3127<br>1612<br>1479<br>3641

Table S3: Electronic absorption spectra of *Veronica Cinerea* Calculated at TD-DFT/ B3LYP/ 6-31g (d,p).

Name	Excited state	Wavelength (nm)	Excitation energy (eV)	Configurations Composition	Oscillator strength
Beta-amyrin	$S_0 \rightarrow S_1$	206.27	6.010	(0.1157) H-2→L,<br>(0.6850) H→L	0.2402
	$S_0 \rightarrow S_2$	190.62	6.504	(-0.11050) H-4→L,<br>(0.63601) H-2→L,<br>(0.235590) H-1→L,<br>(-0.01108) H-4→L	0.0331
	$S_0 \rightarrow S_3$	188.15	6.589	(0.4072) H-3→L,	0.0002

				(-0.1981) H-2→L, (0.5317) H-1→L	
Beta-amyrin acetate	$S_0 \rightarrow S_1$	221.12	5.6070	(-0.13041)H-15→L, (0.15829)H-11→L, (-0.16989)H-10→L, (0.53140)H-9→L, (0.23028)H-8→L	0.0029
	$S_0 \rightarrow S_2$	194.42	6.3770	(0.68337)H→L+1	0.3549
	$S_0 \rightarrow S_3$	160.65	7.7174	(-0.11273)H-45→L, (-0.12508)H-36→L, (0.11534)H-16→L, (0.12290)H-14→L, (0.15621)H-12→L, (-0.14950)H-10→L, (0.12509)H-7→L, (-0.17020)H-6→L, (-0.10422)H-4→L, (0.27712)H-3→L, (0.21902)H-2→L, (-0.22805)H-1→L,	0.0263
Isoorientin	$S_0 \rightarrow S_1$	279.50	4.4360	(-0.11461)H-3→L+2, (0.64129)H→L	0.6252
	$S_0 \rightarrow S_2$	237.31	5.4598	(0.11802)H-4→L+2, (0.42474)H-3→L, (0.15215)H-3→L+4, (-0.35159)H-1→L, (-0.12046)H-3→L+2, (0.18815)H→L+1, (0.23394)H→L+2, (0.10609)H→L+4	0.0081
	$S_0 \rightarrow S_3$	227.09	5.4598	(-0.11164)H-4→L, (0.19134)H-3→L, (0.10213)H-3→L+2, (0.18827)H-2→L, (0.20211)H-2→L+1, (-0.20021)H-2→L+4, (0.28775)H-1→L, (-0.30089)H-1→L+1, (-0.17584)H-1→L+4, (-0.16295)H→L+1, (0.24581)H→L+2	0.0167
Luteolin	$S_0 \rightarrow S_1$	352.26	3.5197	(-0.23677) H-1→L, (0.65851) H→L	0.1293
	$S_0 \rightarrow S_2$	314.88	3.9375	(0.10725) H-3→L, (0.64714) H-1→ L, (0.22075) H→L	0.2931
	$S_0 \rightarrow S_3$	314.26	3.9453	(0.68835) H-4→L, (0.11903) H-4→L+1	0.0000
Chrysoeriol	$S_0 \rightarrow S_1$	357.70	3.466	(0.1226) H-4→L, (0.1426)H-1→L, (0.6665)H→L,	0.2422
	$S_0 \rightarrow S_2$	350.58	3.536	(0.6940) H-3→L,	0.0001

	$S_0 \rightarrow S_3$	336.46	3.685	(0.6722) H-1→L, (-0.1422) H→L,	0.0711
Baicalein (Control)	$S_0 \rightarrow S_1$	354.51	3.4973	(0.10792) H-1→L, (0.68918) H→L	0.0245
	$S_0 \rightarrow S_2$	307.68	4.0297	(0.49151) H-3→L, (-0.10415) H-3→L+1, (-0.17893) H-2→L, (0.44115) H-1→L	0.0993
	$S_0 \rightarrow S_3$	305.08	4.0640	(-0.40934) H-3→L, (0.15290) H-2→L, (0.52632) H-1→L	0.1080

Table S4. Toxic prediction of three compounds through Admet SAR and Protox-III Online server.

Phytocompounds	Hepatotoxicity	Carcinogenicity	Immunotoxicity	Mutagenicity	Cytotoxicity	AOT
Beta-amyrin	Inactive	Inactive	Active	Inactive	Inactive	III
Beta-amyrin acetate	Inactive	Active	Active	Inactive	Inactive	III
Isoorientin	Inactive	Inactive	Inactive	Active	Inactive	III
Luteolin	Inactive	Active	Inactive	Active	Inactive	III
Chrysoeriol	Inactive	Inactive	Inactive	Inactive	Inactive	III
Baicalein (Control)	Inactive	Active	Inactive	Active	Inactive	III

AOT: Acute oral toxicity

Supplementary Figure

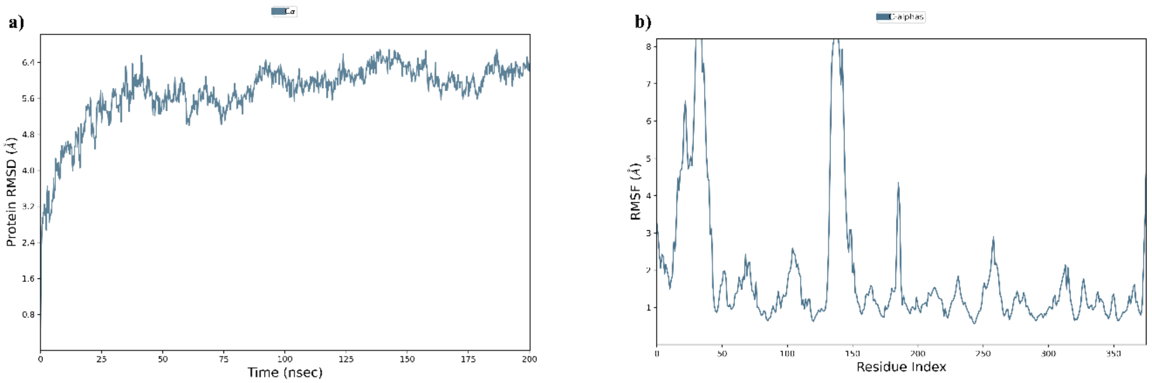

Fig. S1. a) RMSD and b) RMSF plots of NSP1.
