# Supplementary material for: Computational identification of Vernonia cinerea-derived phytochemicals as potential inhibitors of nonstructural protein 1 (NSP1) in dengue virus serotype-2
Source: Front Pharmacol. 2024 Oct 15;15:1465827. doi: 10.3389/fphar.2024.1465827 (PMC11518830; doi:10.3389/fphar.2024.1465827)
Supplement: Supplementary file 1 [file DataSheet1.zip › Supplementary file 1.PDF]

1. Enter the Avogardo-optimized PDB file into the Gaussian program.

2. Right-click on the molecule and set the Gaussian calculation (Ctrl+G).

3. Select the job type as Optimization, and at the method section, select mechanics and UFF (universal force field).

4. Move to the Link0 option, set the memory and processor limit based on the computational power (In our case: Memory 150MW, Processor 4)

5. Move to pop. option and select the level of output at Full

6. Subsequently, move to the solvation option and set default from the model menu and water from the solvent menu.

7. Finally, the job is submitted and saved in Gaussian Input Files(.gjf)
